# Effect of TGFβ1, TGFβ3 and keratinocyte conditioned media on functional characteristics of dermal fibroblasts derived from reparative (Balb/c) and regenerative (Foxn1 deficient; nude) mouse models

**DOI:** 10.1007/s00441-018-2836-8

**Published:** 2018-04-10

**Authors:** Joanna Bukowska, Marta Kopcewicz, Anna Kur-Piotrowska, Anna Z. Szostek-Mioduchowska, Katarzyna Walendzik, Barbara Gawronska-Kozak

**Affiliations:** Institute of Animal Reproduction and Food Research, Polish Academy of Sciences, Tuwima 10, 10-748 Olsztyn, Poland

**Keywords:** Dermal fibroblasts, Foxn1, Skin, TGFβ, Wound healing

## Abstract

Skin injuries in mammals are healed through repair or regeneration. Our previous studies demonstrated that deficient expression of the transcription factor Foxn1 in epidermis of nude mice accounts for their skin’s pronounced regenerative properties. Since homeostasis within the skin depends on complex interactions between the epidermal and underlying dermal layers, the present study characterizes and compares isolated dermal fibroblasts (DFs) between regenerative nude (Foxn1 deficient) mice and their wild-type Balb/c counterparts. Nude DFs exhibited a higher cumulative number of population doublings (cumulative PD) at low seeding density and increased adipogenic differentiation capacity relative to their Balb/c DF counterparts. Nude DFs displayed reduced migration and gel contraction, functional features associated with wound healing. The comparison of transforming growth factor β family (TGFβ) expression showed significantly higher levels of *Tgfβ3* transcript between nude and Balb/c mice but no differences were detected for *Tgfβ1*. Nude DFs were specifically sensitive to the presence of the pro-regenerative TGFβ3 isoform, showing increased collagen I deposition and alpha smooth muscle actin expression. Viability of Balb/c DFs was stimulated by keratinocyte conditioned media (KCM) from Balb/c (Foxn1 active) but inhibited by nude (Foxn1 deficient) KCM. In contrast, nude DFs did not respond to either KCMs with respect to their metabolic activity. Collectively, the enhanced plasticity and greater sensitivity of nude DFs to TGFβ3 stimulation are indicative of and consistent with their pro-regenerative characteristics. These data support the hypothesis that epidermal Foxn1 plays a critical role in determining the DFs regenerative phenotype.

## Introduction

Fibroblasts, the most abundant cell type within connective tissues, contribute to skin homeostasis and provide critical functions during wound healing. The primary role of dermal fibroblasts (DFs) is to maintain and support skin through the secretion of extracellular matrix (ECM) components (Lorenz et al. 1995). Recent data have demonstrated that the function of DFs is far more complex due to their behavioral, morphological and molecular heterogeneities (Driskell et al. 2013; Lorenz et al. 1995).

Differences in DF features might also contribute to the pattern of wound healing (Lorenz et al. 1995). In mammals, the process of healing occurs with fibrosis and scar formation, a condition characterized by excessive deposition of ECM. Accumulation of the collagen-rich matrix leads to contracture and loss of elasticity and tensile strength of the wounded area. As a consequence, the post-injured tissue often never regains the functionality of uninjured tissue.

Perfect healing in the process of regeneration, characterized by scar-free repair, has been demonstrated in fetuses across many mammalian species including human (Lorenz and Adzick 1993). Scar-free healing has been observed in the skin of adult animals in only two cases: Foxn1-deficient nude mice (Gawronska-Kozak 2011; Gawronska-Kozak et al. 2006) and African spiny mice (*Acomys*) (Seifert et al. 2012). Our previous data revealed substantial differences between DFs isolated from regenerative nude mice and their wild-type C57BL/6 (B6) counterparts. DFs from nude mice showed a higher percentage of cells with stem cell markers (CD117 and Oct3/4) and higher expression of matrix metalloproteinases (Mmps) 3, 9, 13 and collagens I and III, than those derived from B6 wild-type mice (Gawronska-Kozak and Kirk-Ballard 2013). Similarities between DFs isolated from fetal skin during the regenerative period (Ihara et al. 1990) and those from nude mice support the hypothesis that they play a pivotal role in the outcome of healing (Gawronska-Kozak 2011; Gawronska-Kozak and Kirk-Ballard 2013).

Foxn1 is a member of the forkhead/winged-helix class of transcription factors expressed in epithelial cells in both the thymus and the skin (Nehls et al. 1994). A loss-of-function mutation in Foxn1 causes a hairless, nude phenotype in mice, rats and human (Nehls et al. 1994). Foxn1 action in the skin contributes to normal keratinocyte growth and differentiation as well as hair development. Furthermore, Foxn1 acts as a regulator of the skin wound healing process through its involvement in re-epithelization and in the epithelial to mesenchymal transition (EMT) process during the early stage of skin wound healing (Gawronska-Kozak et al. 2016).

Interaction between the epidermal layer and its underlying dermis is essential for skin homeostasis. This communication is mediated through the secretion of soluble factors, among which the transforming growth factor β family (TGFβ) exerts a leading role (Rolfe et al. 2007). The three isoforms of TGFβ (1, 2, 3) participate in wound repair and are involved in a number of pathological events affecting the process of healing (Chen et al. 2005). TGFβs are present in all stages of wound healing. During the inflammatory phase, TGFβ released by platelets attracts and recruits neutrophils and macrophages sequentially into the place of injury. At later stages, TGFβ promotes angiogenesis and fibroplasia, leading to ECM remodeling (Klass et al. 2009). Interestingly, fetal skin wounds during the period of scar-free healing have been found to show lower levels of TGFβ1, which are considered to be a profibrotic isoform and higher levels of TGFβ3, which are reported to be antifibrotic (Chen et al. 2005). Shah et al. demonstrated that application of neutralizing antibody to TGFβ1, 2 (Shah et al. 1994, 1995) and exogenous TGFβ3 delivery (Shah et al. 1995) to the site of injury in adult rat skin significantly improved wound healing outcomes through an apparent regenerative mechanism.

The present study was undertaken to characterize and compare DFs derived from the skin of “regenerative” nude mice with those from their wild-type Balb/c counterparts, whose skin exhibits a scar following wound healing. Cumulative population doublings and adipogenic differentiation abilities were investigated to determine basic differences between nude and Balb/c DFs. We also evaluated the basal expression of TGFβ1 and TGFβ3 in the epidermis and skin tissue on the basis of mRNA (epidermis) and protein (skin) levels. Furthermore, the role of both TGFβ isoforms on the cellular and functional behavior of DFs was explored. In this regard, DF metabolic activity, the ability to collagen gel contraction, migration, alpha smooth muscle actin (αSMA) expression and collagen I production were assessed to describe differences between nude and wild-type DFs. In addition, we used keratinocyte-conditioned media (KCM) collected from Balb/c (Foxn1 active) and nude (Foxn1 deficient) cultures to assess the contribution of epidermal soluble factors.

## Materials and methods

### Animals

Newborn Balb/c (Balb/c/cmdb) and nude (Cby.Cg-Fox1<nu>/cmdb) mice were used in the present study. Animals were bred and housed in temperature- and humidity-controlled rooms (22 ± 2 °C and 35–65%, respectively) with a 12-h light/12-h dark cycle, according to the standards of hygienic category SPF (Specific Pathogen Free) in the Center of Experimental Medicine, Medical University of Bialystok, Poland. Animals were anesthetized by isoflurane, sacrificed and dissected skin samples were transported in PBS to the laboratory. All experimental animal procedures were approved by the Ethics Committee of the Medical University of Bialystok, no. 101/2015.

### Cell isolation and culture

Excised skin tissues from newborn Balb/c and nude mice during five independent experiments (3–5 animals per experiment; total *n* = 20 Balb/c and *n* = 20 nude) were subjected to enzymatic digestion, according to a modified procedure described previously (Zuber et al. 2014; Gawronska-Kozak et al. 2016). In brief, to separate the epidermis from the underlying dermis, tissues were first digested with 6 U/ml dispase I (Life Technologies) overnight at 4 °C. Following this, the epidermis was either collected and stored at − 80 °C until RNA isolation or placed in warm 0.05% trypsin-EDTA solution, digested for 3 min and then filtered through 70-μm strainers for keratinocytes isolation. Next, keratinocytes were collected by a series of three trypsin digestion and filtration and were then centrifuged at 250×*g* for 9 min at room temperature. The pelleted cells were suspended and maintained in keratinocyte seeding medium, which consisted of Dulbecco’s modified Eagle’s medium (DMEM/F-12; Sigma-Aldrich) containing 10% fetal bovine serum (FBS; Life Technologies), 0.2% primocin (InvivoGen) and 120 μM β-mercaptoethanol (Sigma-Aldrich). The remaining tissue was digested for 60 min in 220 U/ml of collagenase type I (Life Technologies) and filtered through 100 μm strainers. Cells were centrifuged at 250×*g* for 9 min. Pellets were re-suspended in culture medium DMEM/F12 supplemented with 15% FBS with antibiotics (penicillin/streptomycin; Sigma-Aldrich) and counted in Countess™ automated cell counter (Invitrogen). The primary cultured keratinocytes and dermal fibroblasts (*p* = 0) were used for further experiments.

### Collection of KCM

Subconfluent keratinocytes (*p* = 0) from Balb/c and nude mice seeded on six-well plates were incubated with 1.5 ml of fresh keratinocyte maintenance medium (CnT basal medium 1 with supplements A, B, C; CELLnTEC). After 48 h of culture, media were harvested, filtered and stored frozen at − 20 °C. The first batches of both types of KCM were taken for further experiments.

### In vitro adipogenic differentiation

DFs from the skin of Balb/c and nude mice at passage 1 were seeded in 24-well plates at a density of 5.0 × 10^4^ cells/well and maintained in DMEM/F12 supplemented with 15% FBS and 1% penicillin/streptomycin, until reaching subconfluency (3 days). Adipogenic differentiation was performed according to the procedure described previously (Gawronska-Kozak 2014). Briefly, subconfluent cells (considered as day 0) were exposed to Adipogenic Medium I containing DMEM/F12, 5% FBS, 1% antibiotics, 0.5 mM isobutylmethylxanthine (IBMX), 1.7 μM insulin and 1 μM dexamethasone for 2 days. For the next 4 days, medium was replaced by Adipogenic Medium II containing DMEM/F12 supplemented with 5% FBS, 1% antibiotic solution, 17 nM insulin (Sigma-Aldrich) and 2 μM thiazolidinedione (TZD; Sigma-Aldrich). All treatments were performed in triplicate. After 6 days in culture, representative cultures were fixed and stained for lipid accumulation with Oil Red O or harvested for total RNA used in real-time polymerase chain reaction analysis of the adipogenic differentiation marker, the adipocyte protein 2 (*aP2*) mRNA expression.

### Oil Red O staining

To detect lipid accumulation, differentiated DFs were fixed for 1 h in 10% formalin at room temperature and stained with a 60% solution of Oil Red O (Sigma-Aldrich) for 10 min. Then, cells were washed several times with water and observed under a microscope (Olympus, IX51) equipped with an Olympus digital camera (XC50). The dye retained by cells was eluted with 100% isopropanol followed by absorbance measurements at 500 nm.

### Cumulative PD

Cumulative population doublings (cumulative PD) were determined in order to monitor DFs growth kinetics during long-term in vitro culture. In brief, DFs from the skin of Balb/c and nude mice at passage 1 were plated in 24-well plates at densities of 5.0 × 10^4^, 1.0 × 10^5^ and 2.5 × 10^5^ cells/well in DMEM/F12 supplemented with 15% FBS and 1% penicillin/streptomycin. After reaching subconfluency, cells were passaged (every 2–4 days) by digestion with 0.05% trypsin and reseeded at concentrations corresponding to the initial number of seeded cells (5.0 × 10^4^, 1.0 × 10^5^, or 2.5 × 10^5^ cells/well). Serial passaging was continued until the harvested cell numbers dropped below the initially plated number. All treatments were performed in duplicate, using cells isolated from five separate pools. The growth kinetics of DFs was calculated by using the following formula cumulative PD = (log_10_ *N*_H_ − log_10_ *N*_I_) / log_10_ (2), where *N*_I_ the initial number of cells and *N*_H_ is the final number of cells at each passage (Cristofalo et al. 2000)*.* The sum of all serially counted cell doublings provided the cumulative PD.

### Cell metabolic activity measurement

DFs from the skin of Balb/c and nude mice at passage 1 were seeded in 96-well plates at a density of 2.0 × 10^4^ per well for 18–20 h. After the cells reached 50–60% confluency, the culture medium was replaced with fresh DMEM/F12 with 2% FBS supplemented with TGFβ1, TGFβ3 (both 10 ng/ml; PeproTech), DMEM/F12 containing 2% FBS (control) or 100% KCM. After 24 h and 48 h of stimulation, cell metabolic activity was measured based on a modified MTT (3-[4,5-dimethylthiazol-2-yl]- 2,5-diphenyltetrazolium bromide; Sigma-Aldrich) colorimetric method. In brief, 10 μl of sterile MTT solution (5 mg/ml) was added to the each well and incubated for 4 h. After incubation, medium was removed and formazan crystals were dissolved in 100 μl of DMSO within 2 h. Absorbance was measured at 570 nm using a microplate reader (ASYS Hitech GmbH, UVM340, Biogenet) and MicroWin 2000 software. All treatments were performed in triplicate, using cells isolated from four separate pools. Cell metabolic activity was calculated as a percentage of control cell viability, which was considered to be 100%.

### In vitro wound migration assay

A scratch assay was carried out according to the method performed by Gawronska-Kozak and Kirk-Ballard (Gawronska-Kozak and Kirk-Ballard 2013). First, we determined the effectiveness of mitomycin C in cell division blockage. DFs from Balb/c mice were seeded in 6-well (at density of 1.9 × 10^5^ per well) and 96-well (at density of 2.0 × 10^4^ per well) plates. After 24 h, the cells were treated with mitomycin C (10 μg/ml; Sigma-Aldrich) for 3 h and cultured in DMEM/F12 supplemented with 5% of FBS for the next 38 h. The numbers of collected cells were determined using hemocytometer (6-well plate) or by MTT metabolic assay (96-well plate). For wound migration assay, DFs from Balb/c and nude mice at passage 1 were plated in 12-well plates at a density of 2.5 × 10^5^ per well for 45 h, when cells reached subconfluency. Then, to prevent cell proliferation, fibroblasts were incubated for 3 h with mitomycin C (10 μg/ml) in DMEM/F12 supplemented with 0.2% of FBS. The cell monolayers were wounded by scratching in a straight line throughout the center of the entire 12 well plate with a 200-μl pipet tip. Debris was removed by washing the cells with PBS. Then, cultures were treated with DMEM/F12 containing 5% FBS, TGFβ1 (10 ng/ml), or TGFβ3 (10 ng/ml); a solution of 5 mM citric acid, which had been used as a diluent for the lyophilized TGFβ, served as a negative control. All treatments were performed in duplicate, using cells isolated from four separate pools. Images were captured with an Olympus microscope (IX51) equipped with an Olympus digital camera (XC50) and analyzed with ImageJ (SciJava software, National Institutes of Health; NIH). Four representative images of scratched areas were photographed and the distance between the unclosed edges was measured. The distance of scratch closure at 0 h was considered to be 100%. The scratched areas were monitored until closure (at 0, 3, 6, 12, 24, 35 and 48 h time points).

### Collagen gel contraction assay

Three-dimensional collagen gels were prepared based on a protocol described by Kobayashi et al. (2005). In brief, cell suspensions (6.0 × 10^4^ cells/400 μl) of DFs from Balb/c and nude mice at passage 1 were mixed in 200 μl of a solution of cold rat tail tendon collagen type I (5 mg/ml; Cultrex). Next, 500 μl of the mixture was added to each well of a 24-well plate, neutralized with 4 μl of 1 M NaOH and allowed to polymerize at room temperature for 20 min. After solidification, gel matrices were overlaid with 500 μl of fresh DMEM/F12 containing 0.1% FBS and antibiotics (control), DMEM/F12 with 10% FBS supplemented separately with TGFβ1, TGFβ3 (10 ng/ml), or KCM. The floating gels were incubated at 37 °C in 5% CO_2_ for 9 days. All treatments were performed in duplicate, using cells isolated from three separate pools. Collagen lattices were photographed with Molecular Imager® Gel Doc™ XR+ Imaging System (Bio-Rad), software ImageLab 4.1 and the areas were measured with ImageJ (SciJava software; National Institutes of Health; NIH). Gel contraction was calculated as the percentage of the initial gel area at time 0, which was considered to be 100%.

### Protein isolation and Western blot analysis

Frozen skin samples from Balb/c and nude mice were crushed in liquid nitrogen. Subconfluent (60–70% confluency) cultures of Balb/c and nude DFs at passage 1 were incubated with DMEM/F12 containing 2% FBS (control), DMEM/F12 with 2% FBS supplemented with TGFβ1 or TGFβ3 (both 10 ng/ml) for 4 days. The collected samples were homogenized in volumes of 200 μl (cells) or 500 μl (skin) RIPA buffer containing protease inhibitor cocktail (Phos Stop Roche, Protease Inhibitor Sigma-Aldrich) and further sonicated with Sonics Vibro-Cell ultrasound sonicator (3 × 20 s, 20 kHz). Protein concentration was measured by the infrared (IR)-based protein quantitation method using a Direct Detect® Infrared Spectrometer (Merck). Briefly, 2 μl of the sample in RIPA buffer was spotted in duplicate onto a hydrophilic polytetrafluoroethylene (PTFE) membrane card and analyzed by a mid-infrared (MIR) spectrometer with the absorption of radiation in the approximate range 4000–400 cm^−1^. Protein quantification was measured based on integration of Amide bonds using directly searchable absorptions on the spectrum curve. Thirty-five micrograms of proteins were separated on 12% SDS-polyacrylamide gels and transferred onto polyvinylidene difluoride membranes (Millipore). The membranes were incubated separately with anti-αSMA (1:1000, rabbit polyclonal, Abcam), anti-TGFβ1 (1:500, rabbit polyclonal, Biorbyt), anti-TGFβ3 (1:100, rabbit polyclonal, Abcam), or anti-GAPDH (1:2500 mouse monoclonal, Abcam) antibodies followed by fluorescent secondary antibodies IRDye 800 (1:5000 goat anti-rabbit, Rockland) and Cy5.5 (1:10000, goat anti-mouse, Rockland). Bands were visualized using the Odyssey imaging system (LI-COR Bioscience) according to the manufacturer’s protocol. Densitometric protein analysis was performed using the ImageJ (SciJava software, National Institutes of Health; NIH).

### ELISA of collagen type I

DFs from Balb/c and nude mice at passage 1 were plated in 24-well plates at a density of 4.5 × 10^5^ per well in DMEM/F12 containing 2% of FBS. When cells reached subconfluency, cultures were treated with the following: TGFβ1 (10 ng/ml), TGFβ3 (10 ng/ml), or 5 mM citric acid (as a negative control). Conditioned media were collected after 24 and 48 h of stimulation and stored at − 20 °C for analysis of collagen concentration. All treatments were performed using cells isolated from four separate pools of skin. Concentrations of collagen I in the culture media were determined using the Enzyme-Linked Immunosorbent Assay Kit for Col1a1 (Cloud Clone Corp.) according to the manufacturer’s protocol. The standard curve for Col1a1 ranged from 0.312 to 20 ng/ml. The intra- and inter-assay coefficients of variation (CV) were < 10 and < 12%, respectively. The sensitivity of the assay was 0.124 ng/ml. Collagen type I concentration was calculated as a percentage of the protein content in the control (untreated) cells cultures, which was considered to be 100%.

### RNA isolation and real-time PCR

Total RNA was purified from skin samples and cell cultures using the TRIzol**®** Reagent (Thermo Fisher Scientific). The RNA concentration and quality were determined spectrophotometrically using an ND-1000 spectrophotometer (NanoDrop Technologies) and agarose gel electrophoresis. Genomic DNA was removed from RNA samples using the DNase I Amplification Grade kit (Sigma-Aldrich). Total RNA (1000 ng) was reverse transcribed to cDNA using a High-Capacity cDNA Reverse Transcription Kit with RNase Inhibitor (Applied Biosystems, MA, USA) according to the manufacturer’s protocol. Real-time PCR for *adipocyte protein 2* (*aP2*) and *cyclophilin* (chosen as a housekeeping gene) mRNA levels was performed using the following primer-probe sequences: *aP2* GCGTGGAATTCGATGAAATCA (forward), CCCGCCATCTAGGGTTATGA (reverse), GCTCTTCACCTTCCTGTCGTCTGCG (probe); *cyclophilin*: GGTGGAGAGCACCAAGACAGA (forward), GCCGGAGTCGACAATGATG (reverse), ATCCTTCAGTGGCTTGTCCCGGCT (probe; Metabion International AG) (Rim et al. 2004). Endogenous mRNA expressions for *Tgfβ1*, *Tgfβ3* and *Hprt1* (chosen as a housekeeping gene), isolated from the epidermis and skin samples of newborn mice, were measured with *Tfgβ1* (Mm01178820_m1), *Tgfβ3* (Mm00436960_m1) and *Hprt1* (Mm01545399_m1) TaqMan® Gene Expression Assays (Applied Biosystems by Thermo Fisher Scientific). Reactions were performed using an ABI ViiA™ 7 sequence detection system (Life Technologies) with the following conditions: 10 min at 95 °C, 45 cycles of 15 s at 95 °C and 1 min at 60 °C. Data obtained in real-time were normalized on the basis of appropriate housekeeping gene content and analyzed using the Zhao and Fernald method (Zhao and Fernald 2005).

### Statistical analysis

Statistical analyses were performed using the GraphPad PRISM, version 6.02 software (GraphPad Software). Two-way analysis of variance (ANOVA) followed by the Tukey’s multiple comparisons test was used to compare the effect of TGFβs and KCM from nude and Balb/c mice on DF metabolic activity as well as to test the effect of TGFβs on DF migration, collagen synthesis and αSMA protein expression. Data on DF migration and gel contraction were analyzed for significance using a paired Student’s *t* test whereas the expression of TGFβ mRNA and protein in the skin tissues and epidermis were analyzed used unpaired Student’s *t* test. Data are expressed as mean + standard error of the mean. A value of *p* < 0.05 was considered statistically significant.

## Results

### Cumulative PD of DFs from Balb/c and nude mice skin

**Fig. 1:**
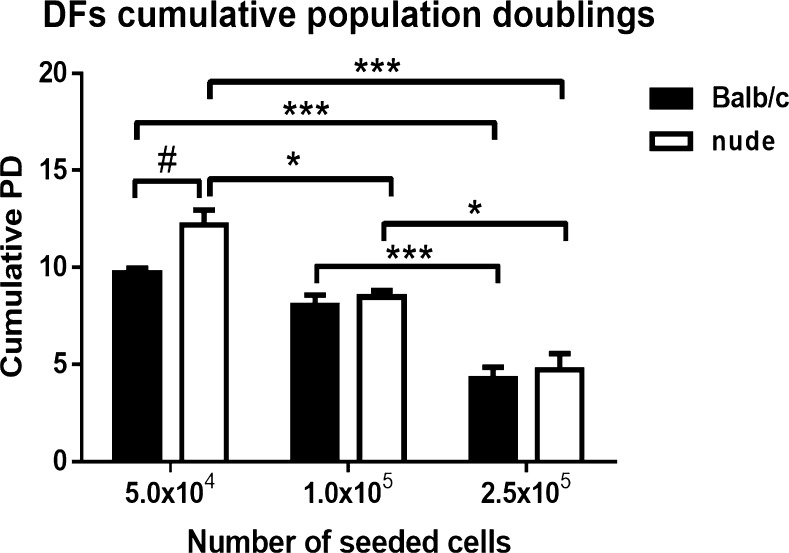
DFs derived from nude mice demonstrate the highest increase in cumulative PD at the lowest cell plating density. Comparison of cumulative population doublings (PD) of DFs derived from the skin of Balb/c and nude mice seeded in 24-well plates at densities 5.0 × 10^4^, 1.0 × 10^5^ and 2.5 × 10^5^ per well. The results are shown as the mean ± SEM. Duplicate wells were used and the experiment was repeated 5 times (*n* = 15 animals; 3 animals per repeat). Asterisks indicate statistically significant differences among DFs seeded at different densities (*p < 0.05; ***p < 0.001)*.* Hashes indicate significant differences between Balb/c and nude DFs seeded at the same density (^#^*p* < 0.05)

For the assessment of potential cellular differences/similarities between Balb/c and nude DFs, we examined cell growth characteristics. The growth kinetics of DFs from Balb/c and nude mice was affected by seeding density (Fig. 1). Analysis of cumulative PD revealed that lower plating density corresponded to the highest cumulative PD in DFs from both Balb/c and nude mice. The growth kinetic pattern of Balb/c DFs showed a significant increase at a density of 5.0 × 10^4^ cells/well when compared to the density of 2.5 × 10^5^ (*p* < 0.001). Similarly, nude DFs showed the highest cell population kinetics at density 5.0 × 10^4^ cells/well relative to the initial cell densities of 1.0 × 10^5^ (*p* < 0.05) or 2.5 × 10^5^ (*p* < 0.001). Moreover, DFs derived from nude mice demonstrated an increased cumulative PD at a density of 5.0 × 10^4^ cells/well compared to their Balb/c counterparts (*p* < 0.05).

### Adipogenic differentiation capacity of DFs from Balb/c and nude mice skin

**Fig. 2:**
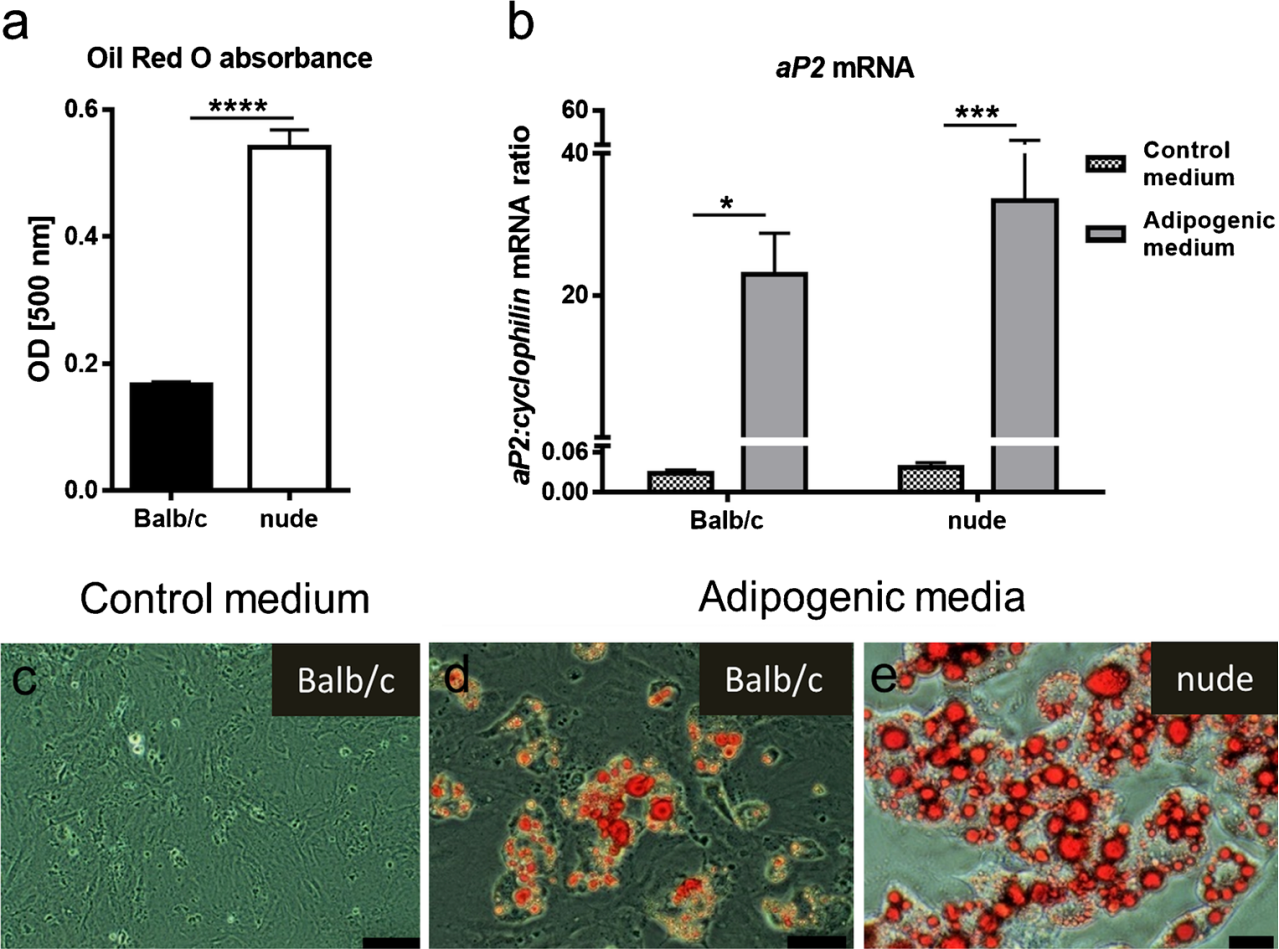
Nude DFs show an increase in adipogenic differentiation potential. Adipogenic capacity of DFs derived from the skin of Balb/c and nude mice seeded in 24-well plates at a density of 5.0 × 10^4^ cells/well was measured by spectrophotometric analysis of Oil Red O staining (**a**) and on mRNA expression levels of adipose tissue marker *aP2* (**b**). Morphology of Oil Red O stained cells cultured with control (**c**) or adipogenic differentiation media (**d**, **e**) within 6 days. The results are shown as the mean ± SEM. Triplicate wells were used, and the experiment was repeated 5 times (*n* = 15 animals; 3 animals per repeat). The asterisks indicate significant differences between groups (*p < 0.05; ***p < 0.001; ****p < 0.0001). Scale bars 100 μm (**c**), 50 μm (**d**) and 20 μm (**e**)

To test the hypothesis that there are substantial differences at the molecular, cellular and functional levels between DFs derived from “scar-forming” mice (Balb/c) and scar-free (nude) mice, we first examined differences in the ability of DFs to differentiate into adipocytes. DFs isolated from the skin of Balb/c and nude mice revealed significant differences in adipogenic differentiation capacity (Fig. 2). Nude DFs showed increased accumulation of lipid droplets measured by spectrophotometric analysis (*p* < 0.0001; Fig. 2a) and histochemical staining of Oil Red O at day 6 of adipogenic differentiation, compared with Balb/c mice (Fig. 2c–e). The mRNA levels of *aP2*, a late marker of adipogenic differentiation, were lower in undifferentiated DFs (cultured in control medium) and elevated following adipogenic stimulation in both Balb/c (*p* < 0.05) and nude DFs (*p* < 0.001; Fig. 2b).

### The expression of TGFβ1 and TGFβ3 in the epidermis and in the skin of newborns

**Fig. 3:**
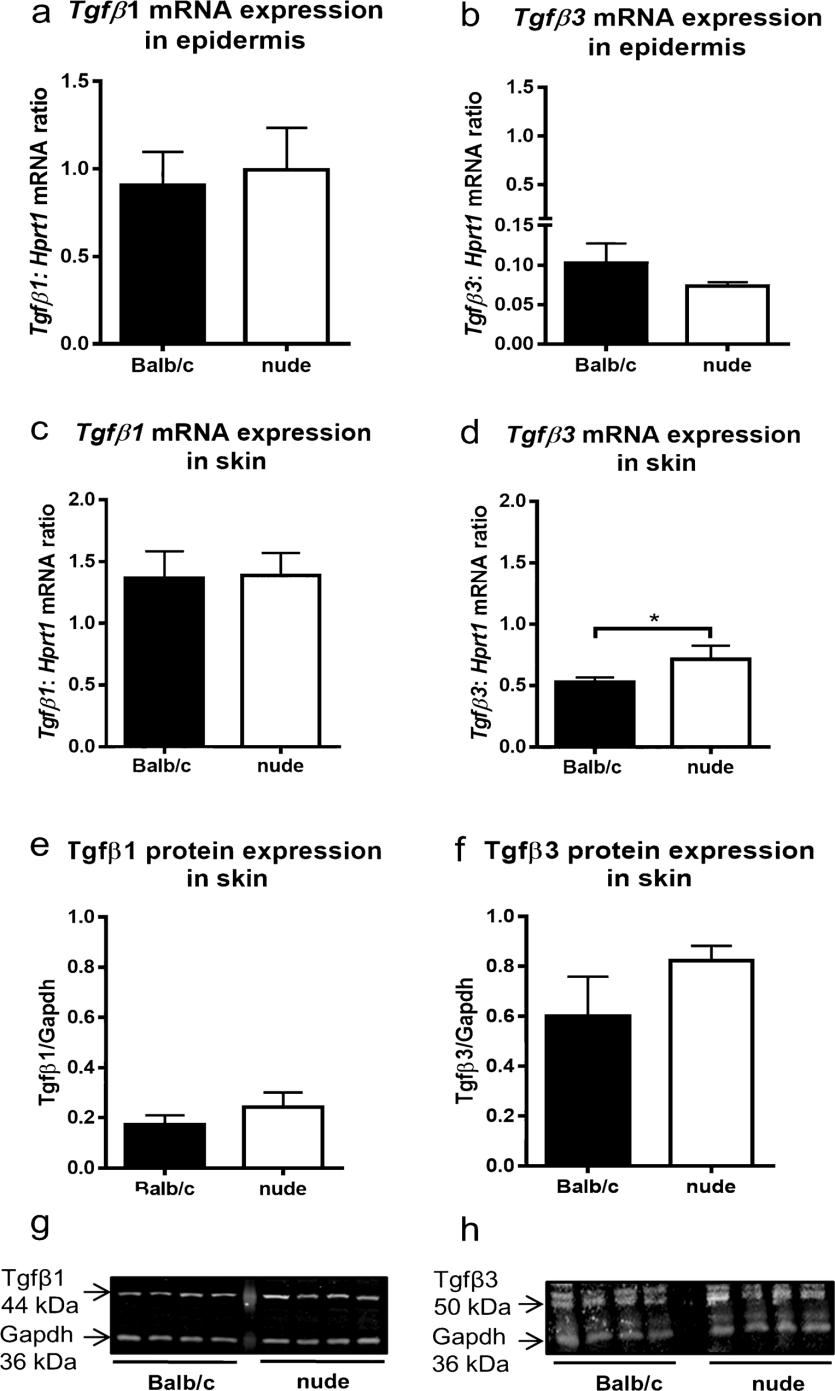
TGFβ1 and TGFβ3 expressions in the epidermis and in the skin tissues of newborn nude and Balb/c mice. Quantitative analysis of *Tgfβ1* (**a**, **c**) and *Tgfβ3* (**b**, **d**) mRNA expression in the epidermis (**a**, **b**) and in the skin (**c**, **d**). Western blots densitometry analysis (**e**, **f**) and representative blots (**g**, **h**) of TGFβ1 (**e**, **g**) and TGFβ3 (**f**, **h**) protein expression in the skin. The results are shown as the mean ± SEM of six animals per group (*n* = 6), each performed in duplicate. The asterisks indicate significant differences between groups (*p < 0.05)

In order to verify whether pro-scarring and pro-regenerative properties of Balb/c and nude skin, respectively, might be related to a specific profile of TGFβs expression, we quantified the levels of *Tgfβ1* and *Tgfβ3* mRNA in the epidermis of Balb/c and nude skin. The analysis of the epidermis showed no differences in *Tgfβ1* and *Tgfβ3* mRNA expression levels between Balb/c and nude mice (Fig. 3a, b). In contrast, the full thickness skin tissue from nude mice exhibited significantly higher levels of *Tgfβ3* transcript when compared to their Balb/c counterparts (*p* < 0.05; Fig. 3d). Western blots quantified by densitometric analysis showed a tendency toward an increased of TGFβ3 protein expression in regenerative nude mice skin relative to Balb/c mice (Fig. 3f, h); however, the difference did not reach statistical significance.

### The effect of TGFβ1, TGFβ3, or KCMs on the metabolic activity of Balb/c and nude DFs

**Fig. 4:**
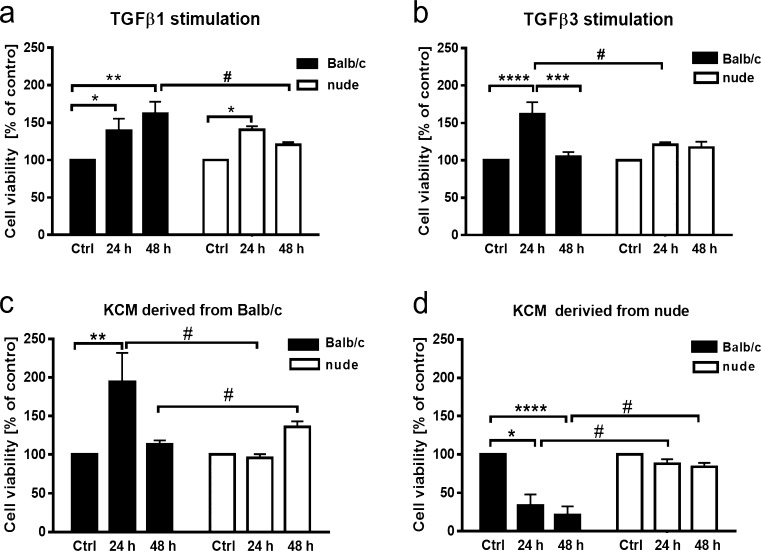
The effect of TGFβ1 (**a**), TGFβ3 (**b**) and KCMs collected from Foxn1 active Balb/c (**c**) and Foxn1 deficient nude (**d**) keratinocytes on viability of Balb/c and nude DFs seeded in 96-well plates at a density of 2.0 × 10^4^ per well. Both TGFβ isoforms (**a**, **b**) increase metabolic activity of Balb/c (black bars) DFs with very limited effect on nude DFs (white bars) viability. Stimulation with KCM collected from Balb/c mice promotes viability of Balb/c DFs (**c**), whereas KCM collected from nude keratinocytes shows an inhibitory impact on these cells (**d**). Both KCMs have no effect of nude DF metabolic activity (**c**, **d**). All values are expressed as percent of control. The results are shown as the mean ± SEM. Duplicate wells were used and the experiment was repeated 4 times (*n* = 12 animals; 3 animals per repeat). The asterisks indicate significant differences relative to the control, non-stimulated cultures (*p < 0.05; **p < 0.01; ***p < 0.001; ****p < 0.0001). Hashes show significant differences between Balb/c and nude DFs (^#^*p* < 0.05)

The effect of TGFβs or KCMs on DFs metabolic activity was assessed by the MTT assay (Fig. 4). Stimulation with TGFβ1 significantly increased the metabolic activity of Balb/c DFs (black bars) within 24 h (*p* < 0.05; Fig. 4a) or 48 h (*p* < 0.01; Fig. 4a). Likewise, a higher viability ratio in TGFβ1 treated cultures was observed in nude DFs (white bars) after 24 h of stimulation relative to the untreated cells (*p* < 0.05; Fig. 4a). However, TGFβ1 showed a stronger effect on Balb/c compared to nude DFs at 48 h of stimulation (*p* < 0.05; Fig. 4a). On the contrary, TGFβ3 had a stimulatory effect exclusively on the growth of Balb/c DFs after 24 h of treatment (*p* < 0.0001) and no effect on nude DFs (Fig. 4b).

Next, we examined the effect of KCMs collected from Balb/c (Foxn1 active) and from nude (Foxn1 non-active) keratinocytes on Balb/c and nude DFs metabolic activity. Stimulation with KCM collected from Balb/c mice promoted the viability of Balb/c DFs within 24 h of culture (*p* < 0.01; Fig. 4c, black bars) but had no effect on the viability of nude DFs (Fig. 4c, white bars). In contrast KCM collected from nude keratinocytes showed an inhibitory impact on Balb/c DF metabolic activity manifested by a 66.07% reduction in absorbance after 24 h of culture (*p* < 0.05; Fig. 4d, black bars) and 78.79% after 48 h (*p* < 0.0001; Fig. 4d, black bars) relative to the control. In contrast, KCM derived from nude keratinocytes had no effect on nude DF expansion (Fig. 4d, white bars).

### Effect of TGFβ1 and TGFβ3 on wound healing in vitro

**Fig. 5:**
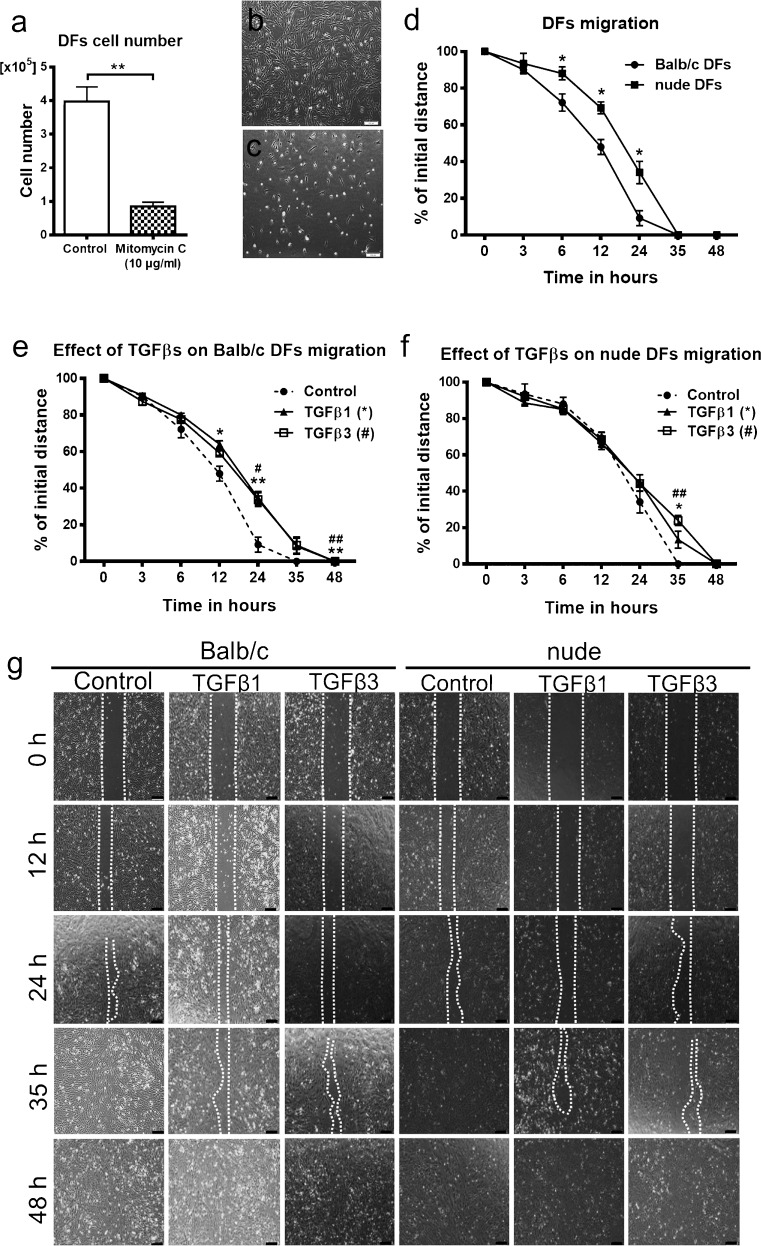
Migratory abilities of Balb/c and nude DFs. Inhibitory effect of mitomycin C (10 μg/ml) on DFs manifested by the decrease in the cell number (**a**) and the cells distribution in the culture plate (**b**, **c**). Wound healing (scratch) in vitro assay (**d**, **e**, **f**, **g**) shows faster migration of Balb/c than nude DFs (**d**). Both TGFβ1 and TGFβ3 isoforms suppress the motility of Balb/c and nude DFs plated in 12-well plates at a density of 2.5 × 10^5^ per well (**e**, **f**, **g**). The results are shown as the mean ± SEM. Duplicate wells were used and the experiment was repeated 4 times (*n* = 12 animals; 3 animals per repeat). The asterisks indicate significant differences between mitomycin-treated and control, untreated DFs **(a)**; Balb/c and nude DFs (**d**); TGFβ1-treated and control Balb/c DFs **(e)**; TGFβ1-treated and control nude DFs **(f)** (*p < 0.05; **p < 0.01). Hashes show significant differences in TGFβ3-treated Balb/c **(e)** or nude (**f**) DFs relative to the control (^#^*p* < 0.05; ^##^*p <* 0.01). Representative images depicting the inhibitory effects of TGFβ1 and TGFβ3 on the migration of Balb/c and nude DFs at different time points (0, 6, 12, 24, 35, 48 h) within 48 h of stimulation (**g**). Scale bars 200 μm (**b**, **c**) and 100 μm (**g**)

The inhibitory effect of mitomycin C on cell division was evaluated (Fig. 5a–c). DFs treated with mitomycin C (10 μg/ml) for 3 h and cultured for an additional 38 h exhibited significantly decreased proliferation based on absolute cell counts (*p* < 0.01; Fig. 5a–c) relative to the untreated control cultures. Since fibroblast migration into the wound bed plays an essential role during wound repair and this early cellular response is mediated by a number of cytokines (Imanishi et al. 2000), we next compared the motility of DFs derived from Balb/c and nude mice and also examined the contribution of TGFβ1 and TGFβ3 in regulating the migratory properties of both types of DFs based on the scratch assay (Fig. 5e–g). To assess the effect of TGFβ1 and TGFβ3 on wound healing by in vitro confluent monolayer of DFs, in which proliferation was blocked by incubation with mitomycin C (3 h, 10 μg/ml), “wounding” was created by scratching in a straight line across the center of each well in a 12-well plate and then the scratched areas were measured until closure. Generally, DFs from Balb/c mice showed significantly faster migration compared to nude mice, based on measurements performed at 6, 12 and 24 h after scratching (*p* < 0.05 for all time points; Fig. 5d, g). Untreated (control) cultures of both Balb/c and nude DFs revealed complete wound closure at 35 h (Fig. 5d, g). Both TGFβ1 and TGFβ3 significantly delayed the motility of nude and Balb/c DFs (Fig. 5e–g). The migratory delay in Balb/c DFs treated with TGFβ1 was detected at 12 h (*p* < 0.05) and 24 h (*p* < 0.01), whereas TGFβ3 inhibition of cell motility was apparent only at 24 h after wounding (*p* < 0.05; Fig. 5e, g). Both cytokines impacted nude DFs at 35 h after wounding (*p* < 0.05 for TGFβ1; *p* < 0.01, for TGFβ3; Fig. 5f, g). At this time point (35 h), cell migration was reduced by 13.38% for cultures treated with TGFβ1 (*p* < 0.05) and 23.09% for cells treated with TGFβ3 (*p* < 0.01), when compared to the untreated control groups (Fig. 5f, g). Moreover, growth factor-exposed cultures showed a delay in wound closure, which was extended to 48 h, in comparison to untreated controls (35 h; Fig. 5e–g). Since DFs were pre-treated with mitomycin C, the observed alterations in wound closure were attributable to changes in migration ability rather than cell proliferation.

### Effect of TGFs and KCMs on collagen gel contraction

**Fig. 6:**
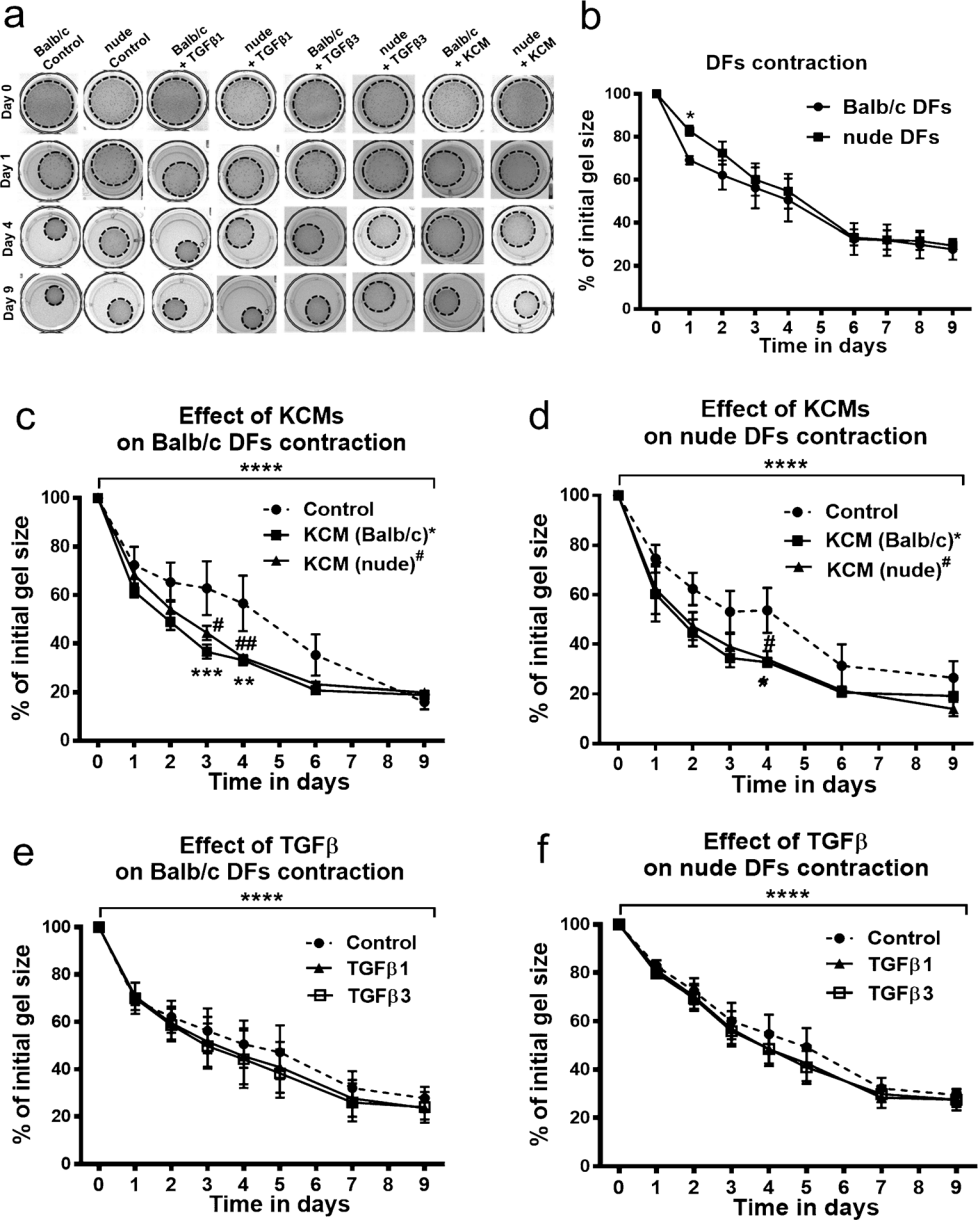
Comparison of contractile abilities of Balb/c and nude DFs seeded at a density of 6.0 × 10^4^ cells per 400 μl of collagen lattices in a 24-well plate. Representative images show the contractile abilities of Balb/c and nude DFs cultured with control medium, KCMs, or with control media supplemented with TGFβ (**a**). DFs were cultured in control (unstimulated) medium, **(b),** KCMs collected from Foxn1 active Balb/c and Foxn1deficient nude keratinocytes (**c**, **d**). DFs from Balb/c (**e**) and nude (**f**) were cultured in medium supplemented with TGFβ1 or TGFβ3. KCMs derived from Balb/c and nude keratinocytes enhance contraction of collagen lattices populated by Balb/c (**c**) and nude (**d**) DFs. Neither TGFβ1 nor TGFβ3 treatment affects matrix size populated by Balb/c (**e**) or nude (**f**) DFs. All values are expressed as percentage of control. The results are shown as the mean ± SEM. Duplicate wells were used and the experiment was repeated three times (*n* = nine animals; three animals per repeat). The asterisks indicate significant differences between Balb/c and nude DFs (**b**); Balb/c (**c**) and nude (**d**) DFs treated with Balb/c derived KCMs relative to the control (*p < 0.05; **p < 0.01; ***p < 0.001). Hashes show significant differences in Balb/c (**c**) and nude (**d**) DFs treated with nude-derived KCM relative to the control (^#^*p* < 0.05; ^##^*p* < 0.01). The asterisks (****p < 0.001) above the graphs –**e**) (**c-f)** show differences between day 0 relative to day 9 (day 0 vs day 9)

To examine the contractile ability of DFs derived from nude (regenerative type of wound healing) and Balb/c (reparative type of wound healing), we performed the collagen gel contraction assay, which is an in vitro model of wound healing and remodeling*.* DFs from both Balb/c and nude mice, embedded within three-dimensional gels composed of type I collagen, reduced gel size over time, regardless of the type of culture medium (control medium, KCMs or medium supplemented with TGFβs; Fig. 6). The gel contraction rate in control media showed similarities between nude and Balb/c DFs with a statistically significant delay detected exclusively at day 1 for nude DFs (*p* < 0.05; Fig. 6b). Replacement of the cell culture medium by KCMs stimulated fibroblast-mediated contraction of collagen gels (Fig. 6a, c, d). Both KCMs, derived from nude and those from Balb/c keratinocytes, significantly promoted contraction of gels by Balb/c DFs at days 3 and 4 (Fig. 6a, c) and by nude DFs at day 4 (Fig. 6a, d). Interestingly, TGFβ1 and TGFβ3 showed no effect on the degree of gel contraction (Fig. 6a, e, f). After 9 days, gels treated with TGFβ1 contracted to 27.75% (Balb/c DFs) and to 29.82% (nude DFs) of their original area (*p* < 0.0001), whereas control gels contracted to 23.53 and 27.55% of their initial size (*p* < 0.0001; for Balb/c DFs and nude DFs, respectively; Fig. 6b, e, f).

### Effect of TGFβ1 and TGFβ3 on DF differentiation into myofibroblasts

**Fig. 7:**
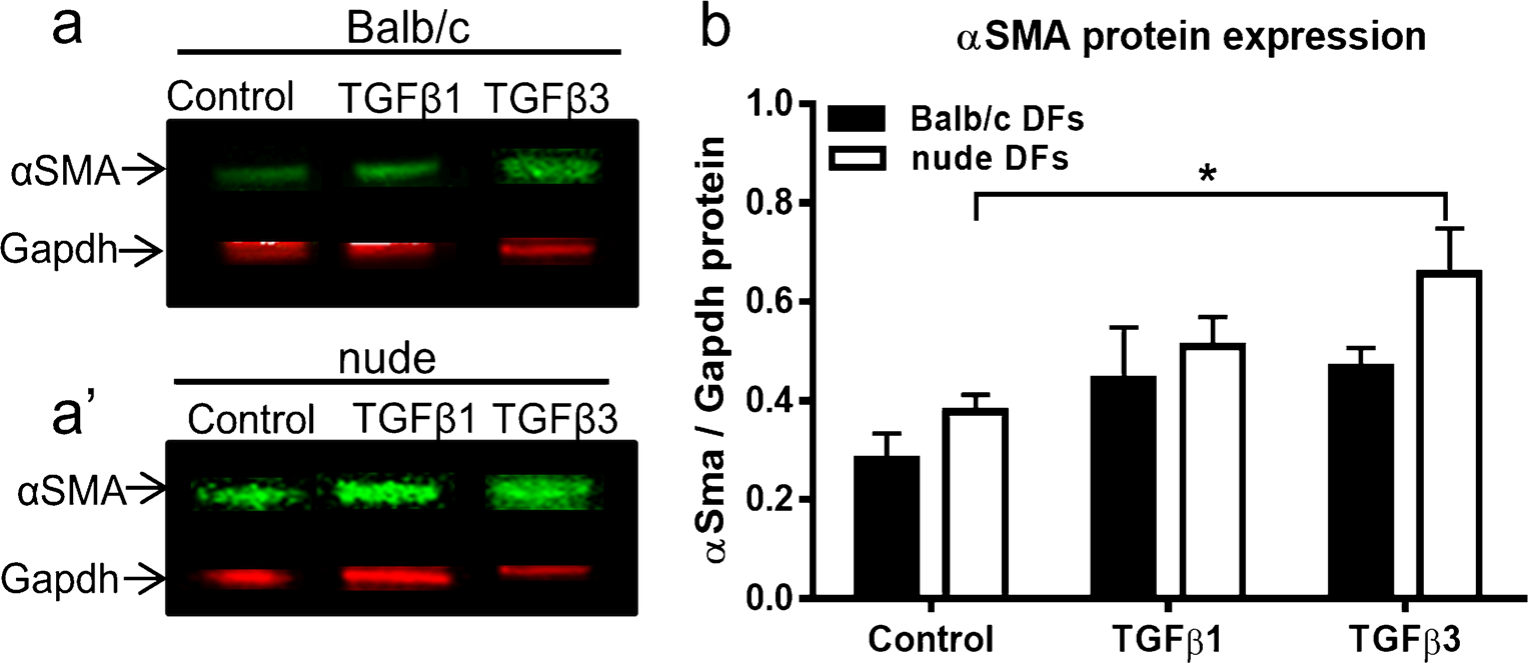
Representative Western blots (a, a’) and densitometry analysis (b) for total αSMA protein content detected in Balb/c and nude DFs. Cells were plated at a density of 2.5 × 10^5^ on 35-mm Petri dishes and stimulated with TGFβ1 or TGFβ3 (10 ng/ml each). Treatment with TGFβ3 increases αSMA expression exclusively in nude DFs (white bars). The results are shown as the mean ± SEM of three animals per group (*n* = 3). The asterisk indicates significant differences between groups relative to the control (*p < 0.05)

The expression of pro-contractile αSMA protein in DFs was analyzed within 4 days of TGFβ stimulation (Fig. 7). Although Western blot assay quantified by densitometric analysis showed a trend toward increased αSMA protein content in nude and Balb/c DFs exposed to TGFβ1 and TGFβ3 treatment, a statistically significant effect was detected only in nude DFs exposed to TGFβ3 (*p* < 0.05; Fig. 7 a, a’, b).

### Effect of TGFβ1 and TGFβ3 on collagen secretion

**Fig. 8:**
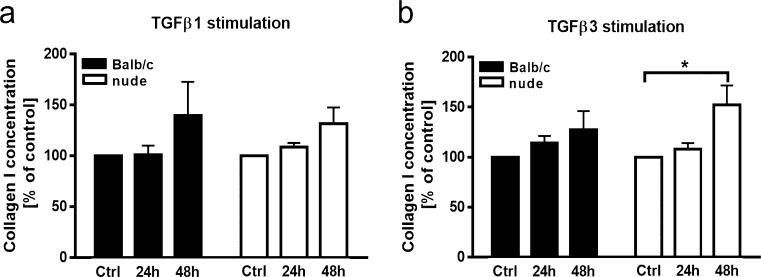
The effect of TGFβ1 (**a**) and TGFβ3 (**b**) (10 ng/ml each) on collagen I protein concentration in the culture media collected from Balb/c (black bars) and nude (white bars) DFs plated in 24-well plates at a density of 4.5 × 10^5^ per well. Exposition to TGFβ3 stimulates nude DFs to collagen I secretion after 48 h of treatment (**b**). All values are expressed as percent of the control. The results are shown as the mean ± SEM. Duplicate wells were used and the experiment was repeated 4 times (*n* = 12 animals; 3 animals per repeat). The asterisk indicates significant differences between groups relative to the control (*p < 0.05)

One of the functions of DFs during wound healing is the production of collagen-rich ECM and this characteristic is modulated by numerous growth factors. We investigated the effect of TGFβ1 and TGFβ3 on collagen I synthesis by nude and Balb/c DFs using the enzyme immunoassay (EIA) method (Fig. 8). While TGFβ3 treatment had a stimulatory effect on collagen I content for nude DFs after 48 h of treatment (*p* < 0.05; Fig. 8b), stimulation by TGFβ1 had no effect on collagen I levels secreted by Balb/c (black bars) or nude DFs (white bars) after either 24 or 48 h of stimulation (Fig. 8a).

## Discussion

The present study shows that (i) DFs from nude mice exhibit enhanced plasticity, comparable to that observed in mesenchymal stem cells, manifested by increased capacity to differentiate into adipocytes and high cumulative PD at low seeding density; (ii) the pro-regenerative capacity of nude mice skin is associated with an increased expression of *Tgfβ3* and a greater sensitivity of nude DFs to the TGFβ3 isoform and; (iii) epidermal Foxn1 plays a critical role in the DF’s phenotype since KCM collected from Balb/c keratinocytes (Foxn1 present) promoted the viability of Balb/c DFs whereas KCM collected from nude keratinocytes (Foxn1 deficient) showed the opposite effect.

Numerous data reported in the literature have demonstrated that seeding density affects the expansion capacity of diverse types of stem cells including human (Colter et al. 2001), mouse (Eslaminejad et al. 2006) and rat (Neuhuber et al. 2008). It has been suggested that low plating densities in mesenchymal stem cells (MSCs) has resulted in higher yields and faster expansion. To some extent, our data related to growth kinetics revealed similarities between MSCs and DFs (especially those isolated from nude mice). Furthermore, to complement the characteristics of DFs, we investigated their adipogenic differentiation capacity. Both types of DFs exhibited the ability to differentiate into adipocytes. However, histochemical analysis showed a significant increase in adipocyte expansion from nude DF cultures, compared to Balb/c, suggesting that regenerative nude phenotype is associated with high levels of cell plasticity.

A study by Whitby and Ferguson (Whitby and Ferguson 1991) demonstrated that scar-free healing observed in mammalian embryos is attributed to the elevated levels of TGFβ3 in the wound area. Shah et al. showed that delivery of TGFβ3 improved restoration of the dermal architecture and reduced scar formation in cutaneous wounds in adult rats (Shah et al. 1995). Our current study showed that the entire skin tissue from nude mice exhibited significantly higher levels of *Tgfβ3* transcript compared to their Balb/c counterparts, suggesting that this cytokine contributes to the pro-regenerative capacity of nude mice skin.

The role of TGFβ1 in wound healing is related to DFs activation and manifested by alterations of their proliferation and viability (Giannouli and Kletsas 2006), migration (Schreier et al. 1993), contraction (Kobayashi et al. 2005), ECM production (Clark et al. 1997) and differentiation into myofibroblasts (Ghosh et al. 2004). In our study, 24 h exposure to TGFβ1 increased the viability of both nude and Balb/c DFs. These data are consistent with the general observation that TGFβ1 increases the in vitro metabolic activity of cells of mesenchymal lineage (Chen and Thibeault 2012). Meran et al., investigating the impact of TGFβ1 on human dermal (non-regenerative) and oral mucosal (regenerative) fibroblasts, found that the cytokine exhibited a distinct proliferative effect (Meran et al. 2008). Whereas TGFβ1 stimulated the proliferation of non-regenerative DFs, it reduced growth of the regenerative oral mucosa cells. In our study, a similar response pattern was observed where upon TGFβ3 treatment increased the metabolic activity of non-regenerative Balb/c DFs while having no effect on regenerative nude DFs.

The monolayer scratch assay revealed that both types of DFs responded to TGFβs stimulation by reduction of their motility. Our data are consistent with those obtained from human foreskin fibroblasts, where exposure to TGFβ inhibited cell migration (Ellis et al. 1992) suggested that TGFβ cytokines, by diminishing cell migration, provide conditions conducive to increased ECM deposition and subsequent fibrosis.

The essential part of the cascade of events associated with the healing processes is the differentiation of fibroblasts into their activated form, myofibroblasts (Darby et al. 1990). Myofibroblasts, characterized by their expression of αSMA, govern wound contraction as well as production of ECM abundant in collagen (Darby et al. 1990). It is well known that TGFβ1 is responsible for fibroblast differentiation and therefore affects αSMA expression (Desmoulière et al. 1993). Rolfe et al. documented that both types of fibroblasts, fetal (scarless) and adult (pro-scarring), differentiated into myofibroblasts; however, this process was faster but more transient in fetal fibroblasts (Rolfe et al. 2007). In our study, αSMA protein expression analysis in DFs cultured in the presence of TGFβ3 indicated an increase in αSMA expression in nude DFs. Moreover, analysis of collagen type I content in culture media collected from nude DFs revealed upregulation of this protein following TGFβ3 but not TGFβ1 stimulation. These collective data showed that nude DFs (regenerative type) are specifically sensitive to the stimulation with the pro-regenerative TGFβ3 isoform.

The cellular differences between nude and Balb/c DFs were further investigated by administration of KCMs from Balb/c (Foxn1 active) and from nude (Foxn1 non-active) keratinocytes. KCM supplementation revealed that it is Balb/c DFs but not nude DFs that are responsive. Whereas KCM collected form Balb/c (Foxn1 active) cultures stimulated Balb/c DF viability, KCM form nude (Foxn1 non-active) keratinocytes had an opposite, inhibitory effect on Balb/c DF metabolic activity. The inability of nude DFs to perceive KCMs might be attributed to their naïve, embryonic characteristics that maintain cell metabolic activity on the constant levels during tissue disturbance and homeostasis disruption. Additionally, the contrasting effects of KCMs collected from Balb/c and nude keratinocytes on Balb/c DF viability implied that the presence or lack of Foxn1 has a considerable impact on the secretome of mouse keratinocytes and might modify metabolic activity of DFs in the underlying dermis. It has been demonstrated that supplementation with KCM influenced cell-cell contacts proliferation (Tenchini et al. 1995) and reduced levels of collagen type I (Ghaffari et al. 2009) in cultured fibroblasts. In fact, the KCM used by other authors (Ansel et al. 1993; Ghaffari et al. 2009; Tenchini et al. 1995) was collected from regular Foxn1 sufficient keratinocytes. Since the composition of nude KCM has not been studied previously, our current findings suggest that the absence of the transcription factor Foxn1 leads to altered keratinocyte paracrine functions, a hypothesis that will be investigated in future studies.

The culture of fibroblasts within three-dimensional collagen matrices in vitro has allowed the determination of cellular function associated with matrix remodeling during wound healing in vivo (Grinnell and Petroll 2010). Our present study has shown that KCM derived from both nude and Balb/c mice but not recombinant TGFβs, was sufficient to stimulate the contraction of collagen matrices populated with Balb/c or nude DFs. Since the range of cytokines and growth factors released by keratinocytes into the culture media is considerable and well documented (Ansel et al. 1993; Ghahary et al. 2001; Wilmer et al. 1994), we postulate that multiple factors regulate the DFs contractile phenotype. Although there were no differences observed in contractile ability between nude and Balb/c KCMs-treated DFs, we did detect a significant delay in gel contraction at day 1 by nude DFs cultured in standard culture medium. Due to the fact that delayed or limited wound contraction is required for induction of regeneration in severely injured tissues (Soller et al. 2012), our study demonstrates that the cellular features of nude DFs are consistent with a pro-regenerative, scarless pattern of healing.

The subtle difference between the nude and Balb/c DF responses to TGFβs reported herein may be insufficient to account fully for the regenerative capabilities of nude skin. Nevertheless, the higher levels of *Tgfβ3* transcript in the skin of nude mice and the greater sensitivity of nude DFs to the presence of TGFβ3 isoform based on the increase in collagen I deposition and αSMA expression are explanatory of their pro-regenerative capacity. In our present study, we examined skin tissues and DFs isolated from newborn mice that may not exhibit a complete pattern of response upon exposure to cytokines due to their immaturity. Therefore, further study is required to explore the contribution of age-related changes in the DFs sensitivity to growth factors stimulation. Tissue resident immune cells may be another possible aspect contributing strain dependent wound healing differences. The mutation in the Foxn1 “nude” gene results in two independent consequences: T cell deficiency and a hairless skin phenotype. Very recent data revealed that Treg lymphocytes localized to the hair follicles (HF) stimulate hair regeneration by promoting the function of HF stem cells (Ali et al. 2017). These findings suggest that the skin-specific, local immune system participates in the host tissue homeostasis. Although experimental evidence supporting a connection between Foxn1 expression and the characteristic of skin immune cells is lacking, we postulate that such a relationship exists and contributes to the maintenance of skin function, the cellular behavior of keratinocytes and DFs and, ultimately, wound healing.

The decision of whether or not healing will result in scar formation is determined during the early phases of healing. Multiple signals including TGFβs and other factors released from the epidermal part of the skin as well as the intrinsic characteristics of DFs govern the core processes that precisely facilitate the selection of one or the other program of wound healing. Our data indicate that the Foxn1 transcription factor contributes to the selective behavioral determinants of DFs; however, additional mechanistic studies will be necessary to elucidate the finer details of Foxn1’s role in connective tissue cell function.
